# De Quervain's Tenosynovitis Virally Exacerbated by SARS-CoV-2 and Influenza Infections: A Case Report

**DOI:** 10.1155/crdi/5117572

**Published:** 2025-01-18

**Authors:** Maria Gergoudis, Logan Laubach, Glenn E. Lee, Jeffrey R. Donowitz

**Affiliations:** ^1^Department of Orthopaedic Surgery, School of Medicine, Virginia Commonwealth University, Richmond, Virginia, USA; ^2^Department of Orthopaedic Surgery, University of California San Francisco-Fresno, Fresno, California, USA; ^3^Department of Orthopaedic Surgery, Virginia Commonwealth University Health System, Richmond, Virginia, USA; ^4^Division of Pediatric Infectious Diseases, Virginia Commonwealth University Health System, Richmond, Virginia, USA; ^5^Division of Pediatric Infectious Diseases, University of Virginia, Charlottesville, Virginia, USA

## Abstract

We present the case of a fully vaccinated 39-year-old male with no pertinent past medical history who initially presented with De Quervain's tenosynovitis which was successfully treated with a corticosteroid injection. His symptoms recurred during a COVID-19 infection, which was treated with a repeat corticosteroid injection. Symptoms recurred during an influenza infection and were subsequently treated with a first dorsal compartment release. The etiology of De Quervain's tenosynovitis remains unclear. It has classically been categorized as a noninflammatory degenerative process, but recent evidence suggests a possible inflammatory connection. Here, we present a case of recurrent De Quervain's tenosynovitis exacerbated by two distinct viral infections. We hypothesize that viral-induced systemic inflammation led to localized recurrence of inflammation within the tendon sheath. Further studies including cytokine analysis and inflammatory markers are needed to advance this hypothesis.

## 1. Introduction

**Figure 1:**
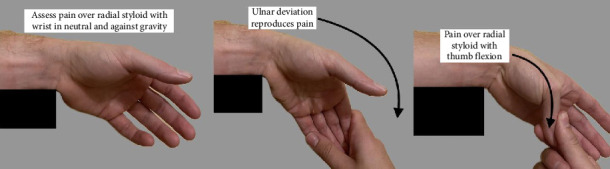
Finkelstein's test.

De Quervain's tenosynovitis is a common disorder characterized by radial-sided wrist pain. Incidence is 10 times higher in women than men and is often associated with overexertion or repetitive movements [1]. It is diagnosed by a history of radial-sided wrist pain and pertinent physical examination findings which include first dorsal compartment tenderness and positive Finkelstein and Eichhoff maneuvers (Figure 1). Neurovascular exam should be normal [2]. A single corticosteroid injection provides complete resolution of symptoms in more than 75% of patients, with few patients requiring a second injection or surgical release [1, 2].

Although De Quervain's is categorized as tenosynovitis, it is not typically classified as an inflammatory condition and has instead been historically characterized by degeneration or mechanical entrapment of the abductor pollicis longus (APL) and extensor pollicis brevis (EPB) within the first dorsal compartment [1]. Thickening of the tendon is proposed to be a result of a mechanical reaction to repetitive stress rather than due to a primary inflammatory insult. This persistent stimulus does not allow the tendon to heal and leads to degenerative tendinopathy [3]. However, recent studies have suggested an increasingly important role of inflammatory mediators in the early stages of tendinopathy [3–5]. Carp et al. found that inflammatory biomarkers such as interleukin-1β (IL-1β), tumor necrosis factor α (TNF-α), and C-reactive protein (CRP) increased with the severity of upper extremity overuse disorders such as wrist sprain/strains, tendinitis, lateral epicondylitis, and bicipital tendonitis. Still, the study did not specifically examine De Quervain's tenosynovitis [6]. Kuo et al. examined retinaculum samples from 13 patients with De Quervain's and provided direct evidence that neutrophil elastase, macrophages, and cyclooxygenase-2 are present in higher quantities in De Quervain tissue versus control specimens [5]. Another study by Kuo et al. found a correlation between the severity of De Quervain's and TNF-α and IL 20 (IL-20) levels and suggested that IL-20 could be a biomarker for De Quervain's [3]. Other than a 2021 retrospective cohort study which concluded that De Quervain's syndrome is associated with an increased risk of Herpes Zoster, no other studies to our knowledge show a correlation between De Quervain's symptoms and systemic infection [7]. This case report demonstrates an association between De Quervain's tenosynovitis and systemic infection with influenza and SARS-CoV-2.

## 2. Case Presentation

**Figure 2:**
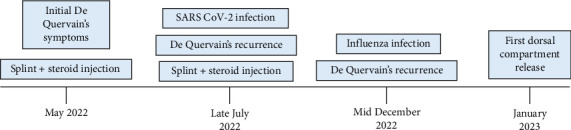
Timeline of events.

The authors certify that this manuscript adheres to CARE (CAse REport) guidelines. Herein, we present an otherwise healthy 39-year-old male physician who presented with dorsal radial wrist pain in early May 2022 (Figure 2). Symptoms began 2 weeks prior upon waking up in an awkward position on an international flight. Symptoms were initially mild but progressed. He felt they were exacerbated by lifting his 1-year-old and 3-year-old children. The exam showed exquisite tenderness over the first dorsal compartment and overlying swelling. Finkelstein and Eickhoff tests were positive. Initial treatment consisted of thumb-spica splinting and corticosteroid injection of 1 mL 1% Lidocaine and 1 mL of 6 mg/mL Betamethasone into the first dorsal compartment sheath. The patient reported full resolution of symptoms within 1 week.

The patient developed a fever on the evening of July 26^th^, 2022, and was diagnosed with COVID-19 by nasopharyngeal PCR on July 27^th^, 2022. The patient denied ever smoking or any history of lung infections such as viral or bacterial pneumonia. The patient was fully vaccinated against COVID-19. The patient was febrile for three days with a recurrence of De Quervain's symptoms within 24 h of fever onset. The patient denied provoking events including lifting his small children. Symptoms began mildly but progressed to severe over the course of several days. The patient returned to the clinic after recovering from his COVID-19 infection with persistent tenderness over the first dorsal compartment and positive Finkelstein and Eickhoff tests. A repeat corticosteroid injection of Lidocaine and Betamethasone was performed, and he similarly had a complete resolution of symptoms within one week (Figure 2).

On December 16, 2022, the patient again developed a fever and malaise. He was diagnosed with Influenza A H3 by nasopharyngeal PCR on December 19, 2022. The patient was vaccinated against influenza in October 2022. Simultaneously, low-grade De Quervain's symptoms returned. His symptoms progressed in severity, and he returned to the office in January 2023 due to the worsening of De Quervain's symptoms. The patient had been wearing the thumb-spica splint since symptom onset and denied exacerbation by lifting his children or other activities of daily living. Due to his having had two previous corticosteroid injections, surgical release of the first dorsal compartment was offered, and he elected to proceed (Figure 2). Intraoperative findings were significant for a thickened first dorsal compartment sheath with a separate EPB sub sheath with two tendon slips. There was a complete resolution of symptoms within 4 weeks of surgery. As of this report's publication date, the patient has had neither a febrile illness nor recurrence of De Quervain's symptoms.

## 3. Discussion

The etiology of De Quervain's tenosynovitis remains unclear with mixed literature over the last several decades. Risk factors for De Quervain's tenosynovitis classically include female sex, manual laborers, new parents, those in the 4th and 5th decades of life, and persons who have frequent repetitive movements such as typing or texting [1]. A meta-analysis in 2023, however, did not show an association between De Quervain's tenosynovitis and repetitive, forceful, or ergonomically stressful manual work [8]. Our patient's onset of De Quervain's was idiopathic while the following two episodes coincided with pyogenic viral illnesses, both of which are known to induce systemic inflammation [9].

A potential link between De Quervain's and viral illness has not been well-explored. Since the description of the disease by Fritz de Quervain in 1895, the inflammatory theory was historically disregarded due to the lack of inflammatory cells on histopathology [3]. Thickening of the extensor retinaculum is a hallmark of the disease which has been attributed to accumulation of mucopolysaccharide consistent with myxoid degeneration, not inflammation [1, 5]. However, recent studies have revealed the presence of inflammatory cytokines in tendinopathies [3, 5]. Inflammation may play a larger role in the early stages of the disease with progression to fibrosis within the tendon as seen with De Quervain's [3]. There is often an interplay between degenerative processes and inflammation with degenerative processes triggered by inflammation [3]. Neutrophil elastase and cyclooxygenase are found in De Quervain's disease retinaculum and correlate with the grade of collagen structure [5]. De Quervain's patients have upregulation of IL-20 receptors and TNF-α correlated with disease severity [3]. This suggests De Quervain's may result from immune activation through macrophages that secrete IL-20 and TNF-α, potentiating disease severity [3].

The correlation between inflammatory cytokines and increased severity of musculoskeletal disorders provides a potential link between viral infection and De Quervain's as in the case of our patient [3, 6]. We hypothesize that viral induced systemic inflammation led to localized recurrence of inflammation in the tendon sheath. The only other documented link between viral infections and De Quervain's tenosynovitis is described in a retrospective cohort study which concluded that De Quervain's syndrome is associated with an increased risk of Herpes Zoster [7]. In the case of this patient, SARS-CoV-2 and influenza A infections preceded recurrence of De Quervain's symptoms. Both SARS-CoV-2 and influenza are highly immunogenic and capable of causing extensive systemic inflammation [9]. Our patient had a fever in both instances signaling a systemic response. This systemic inflammation may have been the trigger leading to the recurrence of De Quervain's symptoms in previously affected tissue. Cytokine or inflammatory marker testing was not clinically indicated; thus none was obtained. Although a separate EPB tendon sub sheath was noted during surgery and is associated with a higher rate of recurrence, there was no mechanical provocation of symptoms, and the precise timing of viral illness preceding De Quervain's recurrence suggests a link [1]. The goal of this case report is to be hypothesis generating, providing the background to encourage experimental rather than observational data. Future studies may benefit from the inclusion of cytokine analysis and inflammatory markers.

Corticosteroid injection and first dorsal compartment release for De Quervain's tenosynovitis are 70%–91% effective [1, 2]. Corticosteroid injections work primarily by decreasing the number of lymphocytes, macrophages, and mast cells, and the high success rate of a single steroid injection to treat De Quervain's supports an inflammatory mechanism of disease [2]. An area of further research may include the potential genetic predisposition to De Quervain's tenosynovitis, which has been briefly described. Individuals with one allele for a particular single nucleotide polymorphism on chromosome 8 were 38% more likely to develop De Quervain's [10]. Though the mechanism is uncertain and requires additional research, this reinforces the idea that the etiology and pathophysiology of De Quervain's may be complex with multiple risk factors. Inflammatory molecules such as IL-6, IL-20, CRP, and TNF-α may be important in the pathogenesis of De Quervain's as with other established inflammatory diseases [3].

## Data Availability

All data generated or analyzed during this study are included in this published article.
